# Ceftriaxone-resistant *Salmonella enterica* serovar Newport: a case report from South India

**DOI:** 10.3389/fpubh.2024.1418221

**Published:** 2024-08-08

**Authors:** Mahadevaiah Neelambike Sumana, Yogeesh D. Maheshwarappa, Morubagal Raghavendra Rao, R. Deepashree, M. V. S. Krishna Karthik, Nikita K. Shah

**Affiliations:** Department of Microbiology, JSS Medical College and Hospital, JSS AHER, Mysuru, India

**Keywords:** *Salmonella* Newport, gastroenteritis, ceftriaxone resistant non-typhoidal *Salmonella*, ceftriaxone resistance, septic shock

## Abstract

*Salmonella enterica* serovar Newport is a human pathogen underreported in most developing countries. It is known for causing gastroenteritis and extraintestinal infections. In this case report, we report the case of ceftriaxone-resistant *Salmonella enterica* serovar Newport from South India, causing acute gastroenteritis in a sixty-year-old female patient having a history of antimicrobial therapy and recent hospital admission. Serovar Newport, especially among antibiotic-exposed patients, poses a significant public health threat due to its ability to acquire multidrug resistance. This emphasizes the necessity for robust surveillance and monitoring of nontyphoidal *Salmonella* infections, particularly given the limited data on serovar Newport in India. Vigilance in clinical practice and public health initiatives is crucial to effectively address the emergence and spread of multidrug-resistant strains.

## Introduction

*Salmonella* is a rod-shaped, motile, Gram-negative, facultatively anaerobic human and animal pathogen belonging to Enterobacteriaceae (1). Salmonellae are non-lactose fermenters that produce catalase (2). Salmonellae contain more than 2,600 serovars, including *S. enterica*. The genus *Salmonella* comprises both typhoidal and non-typhoidal serovars (NTS). The typhoidal serovars that cause human infections are *S*. Typhi, *S*. Paratyphi A, *S*. Paratyphi B, *S*. Paratyphi C. *S*. Typhimurium, and *S*. Enteritidis, which are the most common non-typhoidal serovars (3).

Typhoidal *Salmonella* serovars cause an invasive, life-threatening systemic infection called enteric fever, whereas NTS infections are limited to gastroenteritis (4). Typhoidal *Salmonella* infections are the leading cause of morbidity and mortality. However, food poisoning and enteric infections caused by NTS have emerged as important global health concerns. Approximately 93.8 million human infections and 155,000 fatalities are annually attributed to non-typhoidal *Salmonella* serovars. These serovars contribute to 38% of foodborne illnesses, leading to 35% of hospitalizations and 28% of deaths associated with foodborne diseases (5, 6).

*Salmonella* Newport is an important serovar that causes gastrointestinal epidemics. Additionally, it can lead to extraintestinal infections including neonatal septicemia, chest wall abscess, osteomyelitis, endocarditis, meningitis, and splenic abscess. The risk factors for Newport infection vary based on antimicrobial susceptibility. Young age, international travel, and history of stomach ulcers are associated with drug-susceptible strains of *S*. Newport. In contrast, recent antimicrobial use and the consumption of uncooked food are risk factors for multidrug-resistant strains (7, 8).

*S*. Newport, particularly DT104 (also known as Newport-MDR AmpC) strains as reported, are resistant to ampicillin, chloramphenicol, streptomycin, sulfamethoxazole, and tetracycline. Additionally, Newport-MDR AmpC strains were resistant to amoxicillin/clavulanic acid and cefoxitin and were known for their decreased susceptibility to ceftriaxone. Class-I integrons are most commonly responsible for the clonal spread of ceftriaxone resistance among Newport isolates (9). In developed countries*, Salmonella* Newport is among the top three serovars causing non-typhoidal *Salmonella* infections. The association of *Salmonella* Newport strains with epidemics in India, coupled with antimicrobial resistance, could pose a potential threat to public health. Hence, this case is being reported.

## Case presentation

A 60-year-old woman who has a known case of type 2 diabetes mellitus and poliomyelitis was previously discharged from the orthopedic department and was treated for a fracture of the right femur distal one-third and proximal right-sided tibia (Schatzker type 4). The patient was conservatively treated and discharged for the same. Post-discharge and the following day, the patient presented with chief complaints of vomiting (7–8 episodes, non-bilious, non-blood-tinged, and non-projectile) and loose stools (watery in consistency, non-blood-tinged, or mucoid) for 1 day. She had a history of antimicrobial consumption during her previous admission. The patient was admitted for the above complaints.

On general examination, the patient was febrile with a pulse rate of 106/min, blood pressure was 90/60 mmHg, and SPO_2_ showed a saturation of 91% in room air. The patient had no icterus, cyanosis, clubbing, lymphadenopathy, or edema. A per-abdominal examination revealed epigastric tenderness, and no organomegaly was noted. Respiratory and cardiovascular system examinations were normal. The patient was conscious and oriented. On admission, a provisional diagnosis of acute gastroenteritis in shock with a corrected fractured right femur and tibia was made. In view of the shock caused by gastroenteritis, the patient was empirically started on piperacillin–tazobactam 4.5 g QID to empirically cover Gram-negative infections, doxycycline (injection) 100 mg BD to cover *Vibrio cholerae*, and metronidazole (injection) 500 mg TID to cover protozoans.

Hematological parameters revealed an increase in the total leukocyte count to 22,660 cells/cu mm, with a 93.6% predominance of neutrophils and an increase in the ESR of 90 mm. Her hemoglobin level was 8.9 g/dl. Her bilirubin level was elevated to 1.48 mg/dl. In the blood, there were decrease in the serum albumin and total protein levels of 2.5 g/dl and 4.6 g/dl, respectively. Serum electrolytes and liver function tests were normal. Arterial blood gas analysis revealed severe metabolic acidosis. Her HIV status was negative. As the patient presented with fever and features of gastroenteritis, she was examined to rule out the causes of tropical fever such as enteric fever, dengue, leptospirosis, scrub typhus, and malaria. Serum samples were sent for tropical fever panel analysis, including dengue NS1 and IgM antibodies, Weil–Felix, malarial lactate dehydrogenase antigen, and Leptospira IgM, and the test results came negative. The procalcitonin value was elevated to 22.67 ng/dl, indicating a possible bacterial infection. Abdominal ultrasonography revealed circumferential long segment wall thickening of the colon (8 mm) and cholelithiasis. Refer to Table 1 for additional details.

**Table 1:** Physical and systemic examinations and radiological and laboratory findings.

**Physical examination**	
**Parameter**	**Observation**
Blood pressure	90/60 mmHg
Pulse rate	106 bpm
SPO_2_	91% at room air
Febrile status	Afebrile
**Systemic examination**	
Cardio vascular system	S1 and S2 +
Respiratory system	B/L NVBS (bilateral normal vesicular breath sounds)
Per-abdomen examination	Soft epigastric tenderness+, BS+ (Blumberg's sign), and no organomegaly
Central nervous system	Conscious, oriented, neurocognitive function non-decline, moves all four limbs.
**Radiological findings**	
USG abdomen and pelvis	No ascites, circumferential long segment large bowel wall thickening noted involving the entire colon with a maximum thickness of 8 mm with maintained gut structure. The rest of the bowel loops were normal.
**Laboratory investigations**	
Alkaline transaminase	
Serum glutamic oxaloacetic transaminase (SGOT)	13 U/L↑
SGPT (Serum glutamic pyruvic transaminase)	13 U/L↑
Total bilirubin	1.48 mg/dl↑
Erythrocyte sedimentation rate	90 mmHg in 1 h
Peripheral smear (blood picture)	Normocytic normochromic
Leukocytes	Increased in numbers with increased neutrophils
**Microbiological Findings**	
Dengue NS1 antigen detection (ELFA)	Negative
Dengue IgM antibody detection (ELFA)	Negative
Weil–Felix Test	Negative (titer < 1 in 80)
Leptospira IgM ELISA	Negative
Procalcitonin by ELFA (mini Vidas)	22.67 ng/ml↑
Stool microscopy	Plenty of inflammatory cells seen, no RBCs and no parasitic forms observed
Blood culture	No growth after 5 days of aerobic incubation
Stool culture	Isolated *Salmonella enterica* serovar Newport

↑ indicates Procalcitonin levels are elevated.

Urine microscopy and culture results were within normal limits. Stool microscopic examination revealed Plenty of inflammatory cells, no RBCs, and no parasitic forms. Stool culture yielded non-lactose-fermenting colonies on MacConkey agar and green colonies on Hektoen enteric agar. Culture microscopy revealed motile, Gram-negative bacilli. The bacteria produced catalase but not cytochrome oxidase. They reduced nitrate to nitrites and fermented glucose with gas production. The bacteria did not produce indole, urea was not hydrolyzed, but citrate was utilized. The methyl red test was positive, but the Voges–Proskauer test was negative. The Triple Sugar Iron tube produced an alkaline slant and acid but with abundant hydrogen sulfide, thus making differentiation between *Salmonella* Paratyphi B and *Salmonella* Typhimurium challenging. Serotyping was performed using poly(O) antisera, HI, O2, O4, and O9 antisera from *Salmonella*. In serotyping, the isolate showed agglutination with poly(O) antisera and was negative for HI, O2, O4, and O9 antisera. The report was categorized as a *Salmonella* group, along with antimicrobial susceptibility patterns, including minimum inhibitory concentrations, as shown in Table 2.

**Table 2:** Antibiotic susceptibility report.

**Antibiotic**	**MIC**	**Interpretation**
Trimethoprim–sulfamethoxazole	≤ 20	Sensitive
Ampicillin	-	Resistant
Amoxicillin–clavulanic acid	4	Sensitive
Ceftriaxone	≥64	Resistant
Ciprofloxacin	≥4	Resistant

The antimicrobial susceptibility (AST) test was conducted using a Vitek-2 Compact system (bioMérieux, France) and an N-405 card. The isolate exhibited sensitivity to trimethoprim–sulfamethoxazole and amoxicillin–clavulanic acid but was resistant to ampicillin, cefepime, ciprofloxacin, and ceftriaxone. The AST results obtained from the Vitek-2 Compact system were confirmed by the Kirby–Bauer disk-diffusion method. Quality control for both the Vitek-2 and disk-diffusion methods was routinely conducted following standard operating protocols guided by the CLSI. The isolate demonstrated resistance to ceftriaxone in both the Vitek-2 Compact system and the disk-diffusion method. Consequently, the isolate was forwarded to the National Institute of Cholera and Enteric Diseases (NICED) in Kolkata, West Bengal, India, for speciation and serotyping. The NICED report identified the isolate as *Salmonella enterica* serovar Newport with resistance to ceftriaxone.

Once gastroenteritis due to NTS with ceftriaxone resistance was diagnosed, the injection of piperacillin–tazobactam was continued for 9 days, and doxycycline and metronidazole were stopped. The patient improved with piperacillin–tazobactam and was discharged. Procalcitonin could not be repeated due to cost constraints, and the patient responded clinically to IV piperacillin–tazobactam.

While examining the psychosocial history to find the possible source/or cause of infection, the patient reported that she and her family had no history of travel, were non-vegetarians, and stayed at home where they maintained proper personal and environmental hygiene. Psychosocially, the patient reports no stress or mental health issues, ensuring a stable psychosocial environment. There are no known hereditary conditions or genetic predispositions to bowel disease within the family.

To know if the patient had a previous history of antibiotic therapy as the NTS isolated was ceftriaxone-resistant, history revealed that despite being diabetic, the patient does not have a history of recurrent hospitalizations or frequent medical interventions. Her diabetes has been managed effectively. During her previous stay in the orthopedic department, she was treated with cefoperazone–sulbactam 1.5 g IV BD for 5 days. This suggests that the current resistance status of the organism may be related to this, as cefoperazone is also a third-generation cephalosporin. (7, 8) The focus also remains on the potential nosocomial source of infection encountered during her last hospital stay.

## Discussion

Non-typhoidal *Salmonella* (NTS) infections are a significant cause of bacterial foodborne gastroenteritis and are responsible for heightened mortality (10). The mortality rate is associated with increasing multidrug resistance in NTS strains, defined as resistance to trimethoprim–sulfamethoxazole (TMP-SMX), ampicillin, and chloramphenicol. Antimicrobial resistance (AMR) in non-typhoidal *Salmonella* limits therapeutic options and increases the risk of treatment failure (11). Additionally, NTS strains possess biological mechanisms that increase virulence. Hence, close monitoring of NTS infections is warranted (12, 13).

In this case report, *Salmonella enterica* serovar Newport was isolated from a stool sample of a patient diagnosed with acute gastroenteritis in shock. The patient had a history of antimicrobial therapy, which poses significant risk factors for acquiring multidrug-resistant serovar Newport. Additionally, the consumption of uncooked food is another identified risk factor for serovar Newport. The strain showed resistance to ampicillin, ceftriaxone, and ciprofloxacin but showed sensitivity to trimethoprim–sulfamethoxazole and amoxicillin–clavulanic acid. Similar multidrug-resistant serovar Newport strains have been isolated in studies conducted by Kumar et al. and Paudyal et al. (5, 14).

Several studies have highlighted the rise of multidrug resistance (MDR) in *Salmonella* Newport. In 2001, the Centers for Disease Control and Prevention (CDC) reported increased MDR S*almonella* serovar Newport infections. These strains exhibited reduced susceptibility to ceftriaxone (MIC ≥ 16 μg/mL) and were traced back to individuals associated with a daycare on a dairy farm. The CDC underscored the significant impact of antimicrobial overuse on the proliferation of Newport-MDRAmpC strains. Consequently, they stressed the importance of promoting the judicious use of antimicrobials in both human healthcare and animal husbandry as a critical measure to combat this issue (15).

A study conducted by Medalla et al. in the United States reported a notable increase in ceftriaxone resistance (MIC ≥4 μg/mL) in human Newport isolates, from 0% in 1996 to 7.1% in 2009, with a peak of 25% in 2001. This surge in resistance between 1996 and 2001 was attributed to the emergence of ACSSuTAuCx Newport phenotypes, with local dairy cattle identified as a significant reservoir. The subsequent decline in ceftriaxone resistance observed in 2009 may be linked to a reduction in ACSSuTAuCx resistance among serovar Newport cattle. Therefore, implementing the One Health approach strategy is crucial to trace the source of infection and effectively contain the spread of multidrug-resistant Newport isolates (16).

Studies conducted by Lopes et al. and Hoelzer et al. in the US reported infections of multidrug-resistant serovar Newport in both humans and animals (17, 18). Recently, Ford et al. (19) reported multidrug-resistant *Salmonella* serovar Newport infections in patients with a travel history to Mexico. These patients frequently consumed beef products. The authors suggested that the resistant strain could disseminate via travelers, animals, imported foods, and domestic food products. Consequently, they emphasized the importance of adopting safe food and beverage consumption practices while traveling and implementing interventions throughout the food production chain to ensure the safety of beef products and to prevent such infections (19).

Studies by Kumar et al. (5) and Deekshith et al. (20) in India have reported multidrug-resistant NTS serovars. However, very few studies have focused on ceftriaxone resistance, such as ceftriaxone-resistant *Salmonella enterica* serovar Kentucky reported by Neelambike et al. from South India and ceftriaxone resistance in NTS reported by Pragasam et al. Studies conducted by Borah et al. and Taneja et al. have reported ceftriaxone-resistant serovar Newport isolated from human cases in India (21, 22). Additionally, Waghamareet et al. reported ceftriaxone-resistant serovar Newport from poultry farms and processing units (23).

All the above studies emphasized the importance of source tracing and containing the infection in animals as part of a One Health strategy. Additionally, inappropriate treatment and disposal of sewage continue to be major contributors to the dissemination of NTS. Ceftriaxone resistance in non-typhoidal *Salmonella* (NTS) is mediated by plasmid-borne resistance genes such as blaCTX-M-1, blaTEM, and blaCMY (24). Furthermore, IncH12 and IncI1 plasmids are implicated in ceftriaxone resistance (24). The presence of these genes in plasmids poses a serious threat due to their potential for widespread dissemination. The increase in ceftriaxone resistance necessitates the use of azithromycin as an alternative treatment, but emerging resistance to azithromycin in NTS is also being reported. Therefore, there is an urgent need to control the spread of drug-resistant *Salmonella* and curb the overuse of antimicrobial agents to improve the treatment strategies for salmonellosis.

The limitations of the case report include not being able to trace the probable source of infection and not conducting a genomic evaluation of the isolate. Compared to Western countries, the limited data related to serovar Newport from India may be attributed to decreased surveillance. However, it is crucial to monitor non-typhoidal *Salmonella* infections in patients with a history of antibiotic consumption, as they are associated with multidrug-resistant *Salmonella enterica* serovar Newport. This highlights the importance of reporting this case.

## Conclusion

*Salmonella enterica* serovar Newport poses a significant public health concern, particularly in patients with a history of antibiotic consumption. This drug-resistant strain presents challenges in treatment options and underscores the importance of robust surveillance and monitoring of non-typhoidal *Salmonella* infections. The scarcity of data on serovar Newport in India compared to that in Western countries highlights the need for increased attention and research. Close monitoring of antibiotic use and continued surveillance efforts are essential for addressing the emergence and spread of drug-resistant strains. This case report emphasizes the importance of vigilance in clinical practice and public health initiatives to mitigate the impact of ampicillin- and ceftriaxone-resistant *Salmonella enterica* serovar Newport infections.

## Data availability statement

The original contributions presented in the study are included in the article/supplementary material, further inquiries can be directed to the corresponding author.

## Ethics statement

The studies involving humans were approved by JSS Medical College Institutional Ethical Committee. The studies were conducted in accordance with the local legislation and institutional requirements. The Ethics Committee/institutional review board waived the requirement of written informed consent for participation from the participants or the participants' legal guardians/next of kin because it is case report not an experimental study. Written informed consent was obtained from the individual(s) for the publication of any potentially identifiable images or data included in this article.

## Author contributions

MS: Conceptualization, Supervision, Writing – review & editing. YM: Writing – review & editing, Data curation, Formal analysis, Writing – original draft. MR: Supervision, Writing – review & editing, Conceptualization. RD: Supervision, Writing – review & editing. MK: Supervision, Writing – review & editing. NS: Writing – review & editing, Supervision.
